# Supplementary material for: Alzheimer’s Amyloid β Peptide Induces Angiogenesis in an Alzheimer’s Disease Model Mouse through Placental Growth Factor and Angiopoietin 2 Expressions
Source: Int J Mol Sci. 2023 Feb 24;24(5):4510. doi: 10.3390/ijms24054510 (PMC10003449; doi:10.3390/ijms24054510)
Supplement: Supplementary file 1 [file ijms-24-04510-s001.zip › ijms-2189592-supplementary final.pdf]

Supplementary figures:

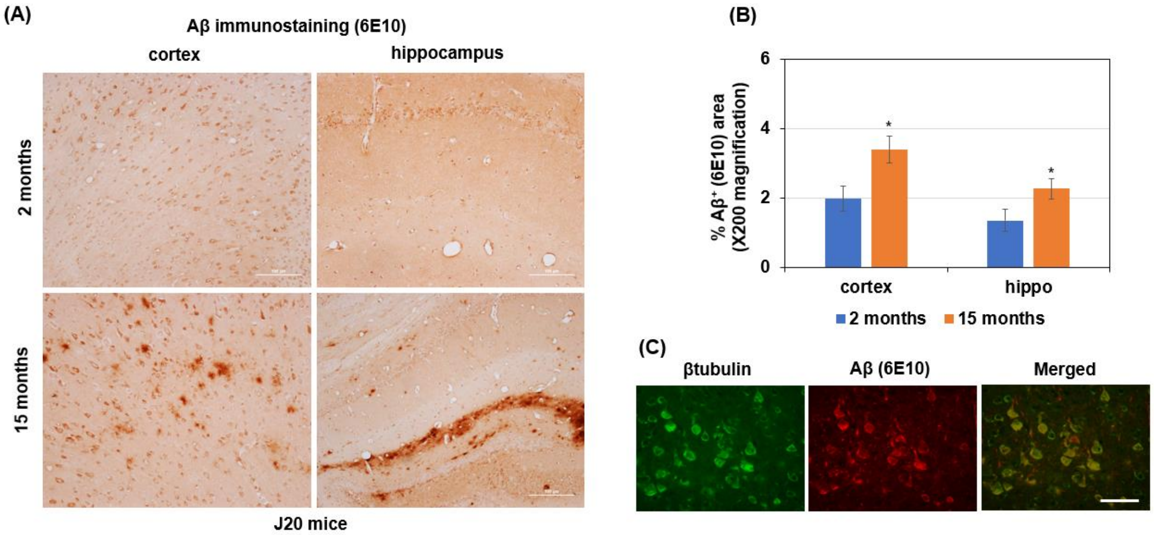

Supplementary Figure S1. Time-dependent changes of Aβ protein deposition in J20 mouse brains. Aβ deposition in J20 mouse brains at 2 months and 15 months of age were evaluated by immunostaining. Representative photomicrographs of the immunostaining are shown in (**A**), where the pictures of the upper row are from 2-month-old mice, and that of the lower row are from 15-month-old mice. The immunostaining photomicrographs were quantified using ImageJ, as described in the Materials and Methods. The quantified data are shown in (**B**). The numerical data presented here as averages ± SD (n= 5 J20 mice at each time point). Statistical significances are denoted as follows: *p< 0.05 vs 2-month-old mice. Bar= 100 µM. (**C**) Aβ expressing cells in the brains of 2-month-old J20 mice were evaluated by double immunofluorescence staining, where βtubulin was used as a marker for neurons. Representative photomicrographs of βtubulin (green), Aβ (red), and their merged picture are shown in (**C**). Bar= 50 µM.

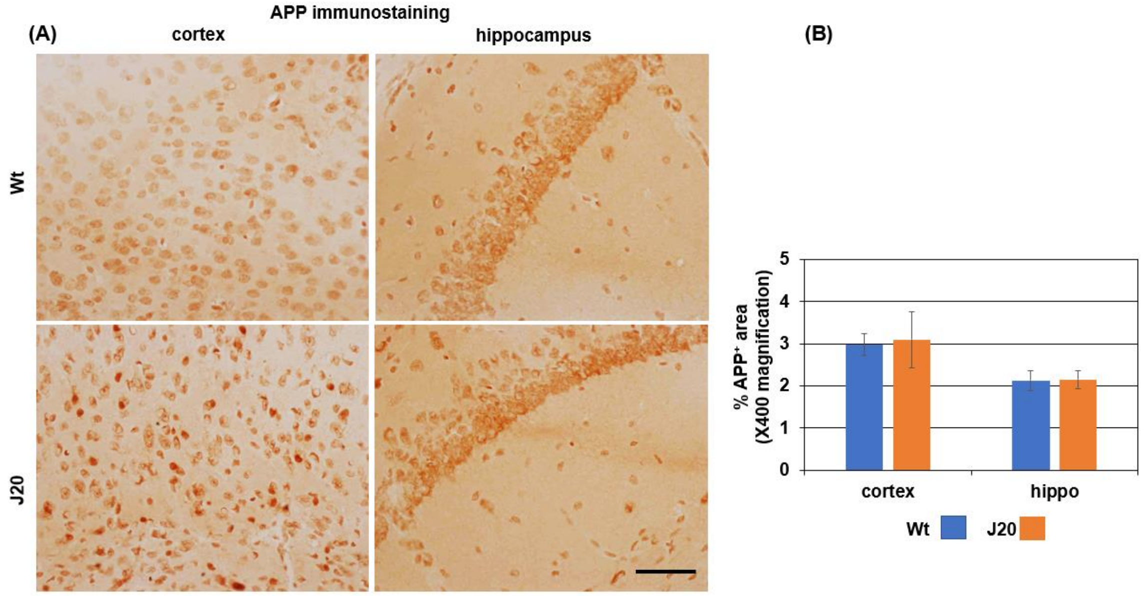

Supplementary Figure S2. Levels of amyloid precursor protein in J20 mouse brains. The levels of amyloid precursor protein in the brains of wild-type (Wt) and J20 mice were evaluated by immunostaining. Representative photomicrographs of the immunostaining are shown in (**A**), where the pictures of the upper row are from 2-month-old Wt mice, and that of the lower row are from 2-month-old J20 mice. The immunostaining photomicrographs were quantified using ImageJ, as described in the Materials and Methods. The quantified data are shown in (**B**). The numerical data presented here as averages ± SD (n= 5 mice at each time point). Bar= 50 µM.

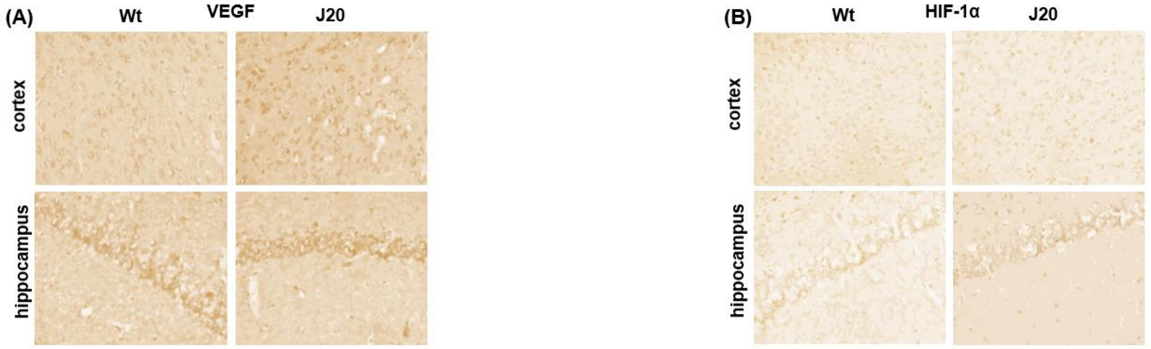

Supplementary Figure S3. Levels of VEGF and HIF-1α proteins in J20 mouse brains. The levels of amyloid precursor protein in the brains of wild-type (Wt) and

J20 mice were evaluated by immunostaining. Representative DAB immunostained photomicrographs of VEGF immunostaining after devonvolution with IHC profiler plagin are shown in (**A**), and that of HIF-1α in (**B**). Wt= Wild-type. Bar= 50 µM.

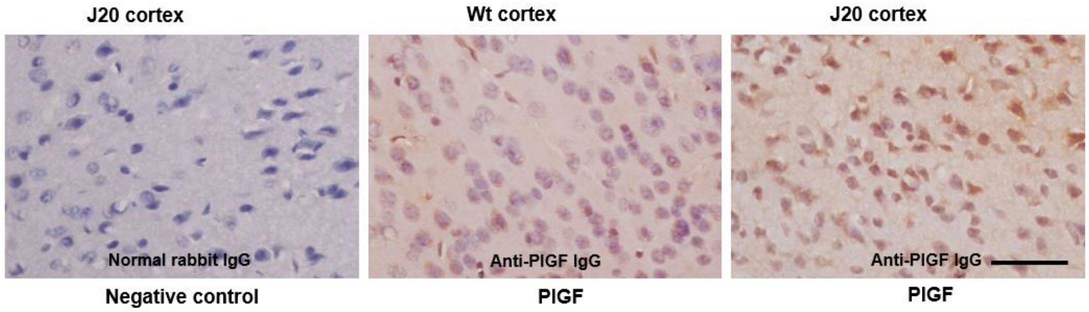

Supplementary Figure S4. Detection specificity of the anti-PlGF antibody. To evaluate the detection specificity of the anti-PlGF antibody, brain tissues of wild-type (Wt) and J20 mice were immunostained with anti-PlGF IgG. Also, a section of J20 brain tissue was immunostained, where normal rabbit IgG was used instead of anti-PlGF IgG (negative control). Representative photomicrographs of PlGF and negative control immunostaining are shown here.

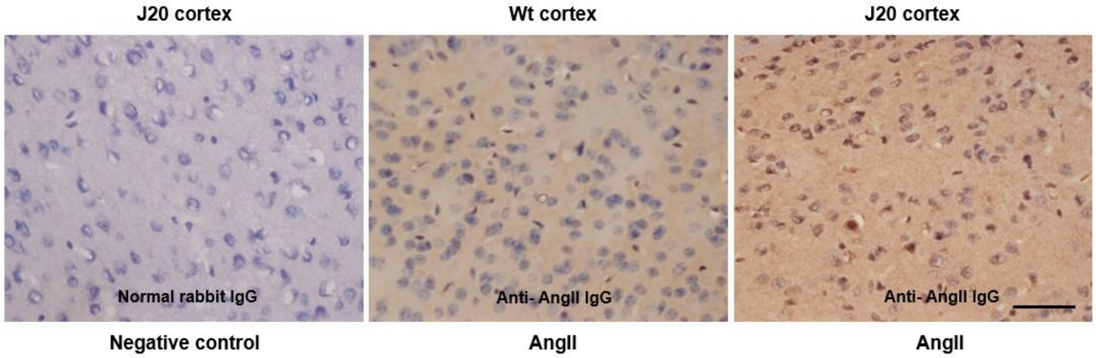

Supplementary Figure S5. Detection specificity of the anti-AngII antibody. To evaluate the detection specificity of the anti-AngII antibody, brain tissues of wild-type (Wt) and J20 mice were immunostained with anti-AngII IgG. Also, a section of J20 brain tissue was immunostained, where normal rabbit IgG was used instead of

anti-AngII IgG (negative control). Representative photomicrographs of AngII and negative control immunostaining are shown here.

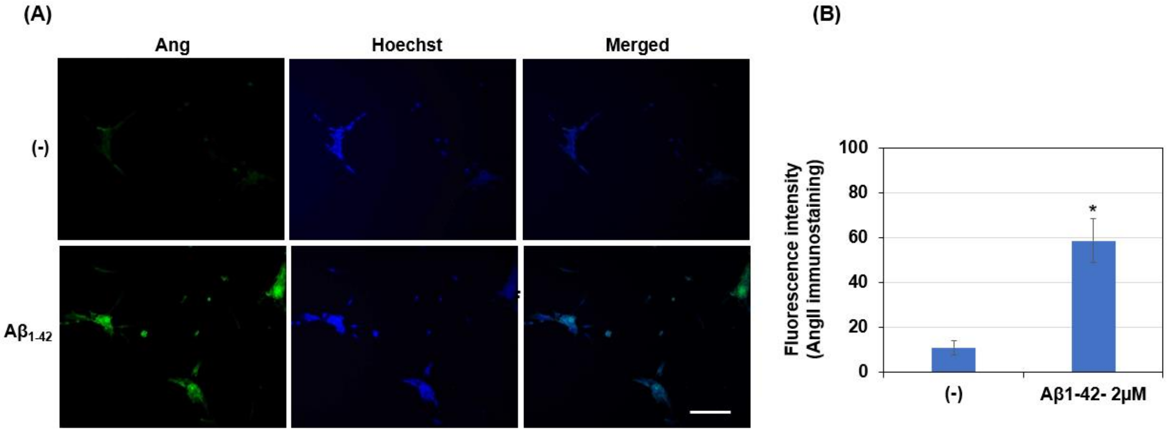

Supplementary Figure S6. Effects of Aβ on AngII protein levels in a neuronal stem cell culture. The levels of AngII were evaluated at protein levels by fluorescence immunocytochemistry after stimulating a neuronal stem cell line (NMW7) with $A\beta_{1-42}$ (2 μM) for 48 h. After stimulation, the cells were stained with anti-AngII IgG, and the immune reaction was visualized with FITC-conjugated anti-rabbit IgG. Nuclei were identified with Hoechst. Representative photomicrographs of (-) and $A\beta_{1-42}$ (2 μM) stimulated NMW7 are shown here (**A**). To quantify AngII levels, the fluorescence intensities were analyzed with ImageJ, as described in the Materials and Methods. The average fluorescence intensities are presented in (**B**). The numerical data presented here as averages ± SD (n=5 experiments). Statistical significances are denoted as follows: $*p < 0.001$ vs medium stimulated (-) condition. Bar= 50 μM.
